# Dexmedetomidine prevents acute kidney injury after adult cardiac surgery: a meta-analysis of randomized controlled trials

**DOI:** 10.1186/s12871-018-0472-1

**Published:** 2018-01-15

**Authors:** Yang Liu, Bo Sheng, Suozhu Wang, Feiping Lu, Jie Zhen, Wei Chen

**Affiliations:** Department of Intensive care unit, Beijing Shijitan Hospital, Capital Medical University, No. 10, Tieyi Road, Haidian District, Beijing 100038 China

**Keywords:** Dexmedetomidine, Acute kidney injury, Cardiac surgery, Meta-analysis

## Abstract

**Background:**

Dexmedetomidine has been shown to confer direct renoprotection by stabilizing the sympathetic system, exerting anti-inflammatory effects and attenuating ischemia/reperfusion (I/R) injury in preclinical studies. Results from clinical trials of dexmedetomidine on acute kidney injury (AKI) following adult cardiac surgery are controversial.

**Methods:**

We searched EMBASE, PubMed, and Cochrane CENTRAL databases for randomized controlled trials (RCTs) comparing the renal effect of dexmedetomidine versus placebo or other anesthetic drugs in adult patients undergoing cardiac surgery. The primary outcome was the incidence of AKI. The secondary outcomes were mechanical ventilation (MV) duration, intensive care unit (ICU) stay and hospital length of stay(LOS), and postoperative mortality (in-hospital or within 30 days).

**Results:**

Ten trials with a total of 1575 study patients were selected. Compared with controls, dexmedetomidine significantly reduced the incidence of postoperative AKI [68/788 vs 97/787; odds ratio(OR), 0.65; 95% confidence interval (CI), 0.45–0.92; *P* = 0.02; I^2^ = 0.0%], and there was no difference between groups in postoperative mortality (4/487 vs 11/483; OR, 0.43; 95% CI, 0.14–1.28; *P* = 0.13; I^2^ = 0.0%), MV duration [in days; *n* = 1229; weighted mean difference(WMD), −0.22; 95% CI, −2.04 to 1.70; *P* = 0.81], ICU stay (in days; *n* = 1363; WMD, −0.85; 95% CI, −2.14 to 0.45; *P* = 0.20), and hospital LOS (in days; *n* = 878; WMD, −0.24; 95% CI, −0.71 to 0.23; *P* = 0.32).

**Conclusions:**

Perioperative administration of dexmedetomidine in adult patients undergoing cardiac surgery may reduce the incidence of postoperative AKI. Future trials are needed to determine the dose and timing of dexmedetomidine in improving outcomes, especially in patients with decreased baseline kidney function.

## Background

Acute kidney injury (AKI) following cardiac surgery is a widely recognized complication in association with high mortality risk [1, 2]. AKI is tightly interrelated with hemodynamic status, inflammatory and nephrotoxic components [3]. Both hemodynamic instability and sympathetic activity during surgery are harmful for renal function [4].Almost half of these patients need mechanical ventilation(MV) support and are related with prolonged intensive care unit (ICU) stay [5, 6].Moreover, along with the increasing high-risk population including advanced age, diabetes mellitus, severe cardiac failure, especially in association with cardiopulmonary bypass, AKI after cardiac surgery has become an interesting and challenge issue in clinical practice [7]. As yet, there is no definite strategy for preventing AKI after cardiac surgery [8].

Dexmedetomidine, a highly selective α2 adrenoreceptor agonist, induces sedation, analgesia, hemodynamic stabilization, anti-inflammation, as well as diuresis [9], and has theoretical advantage for reducing renal injury in animal studies [10, 11]. Several single-center randomized controlled trials (RCTs) with relatively small sample size have addressed this question and the results are controversial [12–14]. Whether perioperative dexmedetomidine could reduce the risk for AKI in adult patients undergoing cardiac surgery remains unclear. In addition, there has been no systematic review that comprehensively focuses on the potential renal effect of dexmedetomidine in adult cardiac surgery. Therefore, we conducted a meta-analysis to evaluate the effect of perioperative dexmedetomidine (compared to placebo or other drugs) on the risk for AKI and mortality.

## Methods

### Search strategy and study criteria

This meta-analysis was performed according to the PRISMA (Preferred Reporting Items for Systematic Reviews and Meta- analyses) guidelines [15] and approved by the Institutional Review Board in Beijing Shijitan Hospital, Capital Medical University. We did a systematic search in PubMed (1999 to March 2017), EMBASE (1999 to March 2017), and Cochrane Library (1999 to March 2017) using the keywords “dexmedetomidine,” “cardiac surgery,” “heart surgery,” “kidney,” and “renal.” English-published RCTs concerning adult patients were included. Exclusion criteria were as follows: emergency surgery, or studies without reporting AKI incidence.

### Literature review and data extraction

The literature review and data extraction were independently completed by 2 investigators (BS and SZW). In case of duplicate records pertaining to a single study, we considered the PubMed database to take precedence. Disagreements were handled by discussion for consensus. Quality assessment was completed using the Cochrane risk of bias tool and Jadad scale. Data extraction included patient characteristics (age, proportion of males, proportion with diabetes, proportion with history of myocardial infarction, proportion with hypertension, baseline left ventricular ejection fraction, baseline creatinine levels, β-blocker use, and statin use), as well as dexmedetomidine dosage.

### Postoperative outcomes

The primary end point was incidence of AKI (defined as RIFLE, AKIN, KDIGO within 7 days after cardiac surgery). Secondary outcomes included all-cause mortality (in-hospital or within 30 days), mechanical ventilation(MV) duration, ICU length of stay, and hospital length of stay(LOS).

### Statistical analysis

For dichotomous outcomes (reported with incidence), we calculated the odds ratio (OR) with 95% confidence interval (CI). For continuous outcomes (reported as mean ± standard deviation, median and interquartile range, or median and range), we calculated mean differences for each study according to the statistical method of Hozo et al. [16] and used weight (the inverse variance of the estimate) to pool the estimate (weighted mean difference, WMD) with 95% CI. We used the random-effect model to pool all the data for the potential clinical inconsistency. Heterogeneity was assessed with the inconsistency statistic (I^2^). Publication bias was assessed by Begg’s test and Egger’s test. *P* < 0.05 (2 sided) was considered to be statistically significant for hypothesis testing. All statistical analyses were performed in REVMAN (version 5.0; Cochrane Collaboration, Oxford, UK) and Stata (version 9.0; StataCorp LP).

## Results

### Study characteristics

**Fig. 1:**
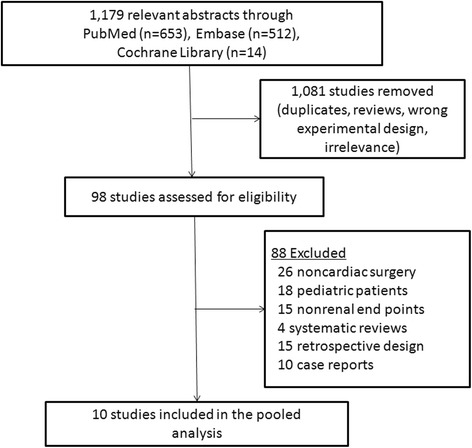
Flow diagram of studies included into meta-analysis

Figure 1 shows the PRISMA flow chart for the RCTs screening and selection process for inclusion in this study. Ten trials enrolling 1575 study subjects ultimately met our criteria (Fig. 1). Two studies were for coronary artery bypass grafting(CABG) [17, 19], seven were for combined cardiac surgery [12–14, 18, 20–22] and 1 was for aortic vascular surgery [23]. Six trials used placebo as control [12, 14, 17, 19, 22, 23], whereas two used propofol [18, 20], one used morphine [21] or remifentanil [13]. Dexmedetomidine was continuously infused at a rate of 0.2 to 0.8μg/kg/h for 24 h after a loading dose (0.4-1μg/kg) in 4 studies [13, 18, 22, 23] or infused at a rate of 0.04 to 1.5μg/kg/h without a loading dose in 6 [12, 14, 17, 19–21].

For postoperative outcomes, AKI incidence was reported in 9 trials [12–14, 17, 19–23], need for dialysis in 1 [18], mortality in 6 [12, 18, 20–23], mechanical ventilation duration in 8 [13, 14, 17–22], ICU stay in 8 [12–14, 17, 18, 20–22], and hospital stay in 6 [13, 17, 18, 20–22].

Study design and patient characteristics were summarized in Tables 1 and 2. The quality assessment was listed in Table 3.

**Table 1:** Summarized Study Design of Included Randomized Trials

Study	Country	Surgery	Dexmedetomidine Dose	Control	Time and Duration of intervention or Control	No. of Patients	Clinical End Point	AKI Definition	Follow-Up
Balkanay2015 I [17]	Turkey	On-PUMP CABG	0.04μg/kg/h-0.05μg/kg/h	placebo	Start preCPB and last for 24 h	31 vs 28	AKI;MV duration; ICU stay; Hospital stay	RIFLE	In hospital
Balkanay2015II [17]	Turkey	On-PUMP CABG	0.04μg/kg/h-0.05μg/kg/h	placebo	Start preCPB and last for 24 h	29 vs 28	AKI;MV duration; ICU stay; Hospital stay	RIFLE	In hospital
Cho 2015 [12]	Korea	Combined	0.04μg/kg/h	placebo	Start immediately after anesthetic induction and last for 24 h	100 vs100	AKI; Mortality; ICU stay;	AKIN	In hospital
DjaianiG 2016 [18]	Canada	Combined	0.4μg/kg 0.2–0.7μg/kg/h	propofol	Start postsurgery and last for 24 h	91 vs 92	AKI; Mortality; MV duration; ICU stay; Hospital stay	NA	In hospital
Leino 2011 [19]	Finland	On-PUMP CABG	0.6 ng/ml	placebo	Start immediately after anesthetic induction and last for 4 h arrive ICU	35 VS 31	AKI; MV duration;	RIFLE	In hospital
Li 2017 [14]	China	Combined	0.1μg/kg/h-0.6μg/kg/h	placebo	Start preCPB and last until the end of MV	142 vs143	AKI;MV duration; ICU stay	KDIGO	30 days after surgery
Liu 2016 [20]	China	Combined	<1.5μg/kg/h	propofol	Start after surgery and last until the end of MV	44 vs 44	AKI; Mortality; MV duration; ICU stay; Hospital stay	AKIN	In hospital
Park 2014 [13]	Korea	Combined	0.5μg/kg 0.2–0.8μg/kg/h	remifentanil	Start after surgery and last until extubation	67 vs 75	AKI; MV duration; ICU stay; Hospital stay	Cr > 100%abovebaseline or new dialysis need	In hospital
Shehabi2009 [21]	Australia	Combined	0.1–0.7μg/kg/ml	morphine	Start within 1 h of adminssin to CICU until the removal of chest drains	152 vs147	AKI; Mortality; MV duration; ICU stay; Hospital stay	NA	12 days after surgery
Ammar 2016 [22]	Egypt	Combined	1 μg/kg over 15 min, followed by 0.5 μg/kg/h	placebo	Start preCPB and last until 6 h after surgery	25 vs 25	AKI; Mortality; MV duration; ICU stay; Hospital stay	NA	30 days after surgery
Soliman 2016 [23]	Egypt	Aortic vascular surgery	1 μg/kg 0.3 μg/kg/h	placebo	Start 15 min before induction maintained to the end of surgery	75 vs 75	AKI; Mortality;	Cr > 115 μmol/L	In hospital

*Abbreviations*: *AKI* Acute kidney injury, *CABG* Coronary artery bypass graft, *CPB* Cardiopulmonary bypass, *ICU* Intensive care unit, *CICU* Cardiac intensive care unit, *MV* Mechanical ventilation, *NA* Not available, *Cr* Creatinine, *RIFLE* Risk–Injury–Failure–Loss–End-stage renal disease, *AKIN* Acute Kidney Injury Network, *KDIGO* Kidney Disease Improving Global Outcomes

**Table 2:** Summarized patient characteristic of the included randomized trials

Study	Age	Male (%)	DM (%)	HP (%)	PreMI (%)	LVEF (%)	CPB duration (min)	Anesthetics	Baseline Serum Creatinine	β-blocker (%)	Statins (%)
Balkanay 2015 I [17]	NA	NA	NA	NA	NA	NA	NA	NA	NA	NA	NA
Balkanay 2015II [17]	NA	NA	NA	NA	NA	NA	NA	NA	NA	NA	NA
Cho 2015 [12]	63	48	19.5	45.5	NA	61.5	131	Sevoflurane	33	NA	63
DjaianiG 2016 [18]	72.55	75.4	21.9	75.4	16.4	NA	98.99	Isoflurane	53	68.85	72.55
Leino 2011 [19]	60.86	89.4	NA	NA	NA	NA	NA	Isoflurane	NA	NA	60.86
Li 2017 [14]	67.18	69.1	32.3	63.2	9.8	NA	102.99	Sevoflurane	69.73	48.42	67.18
Liu 2016 [20]	54.75	39.8	12.5	29.5	NA	65	71.15	Sevoflurane	NA	NA	54.75
Park 2014 [13]	53.81	55.6	9.15	27.5	NA	61.87	166.75	Sevoflurane	NA	NA	53.81
Shehabi2009 [21]	71.25	75.3	29.5	80.1	36.6	NA	98.98	Sevoflurane	NA	NA	71.25
Ammar 2016 [22]	57.25	76	68	82	NA	NA	66.2	Isoflurane	94	56	57.25
Soliman 2016 [23]	58.1	50	30.7	48.7	8.6	52.9	NA	NA	36.67	NA	58.1

*Abbreviations*: *DM* Diabetes mellitus, *HP* Hypertension, *PreMI* Previous myocardial infarction, *LVEF* Left ventricular ejection fraction, *CPB* Cardiopulmonary bypass, *NA* Not available

Values are given as means unless otherwise specified

**Table 3:** Summarized Quality Assessment of Included Randomized Trials

Study	Random sequence generation	Allocation Concealment	Blinding of participants and personnel	Blinding of outcome assessment	Attrition bias	Selective reporting	Jadad scale
Balkanay 2015 I [17]	Yes	Unclear	Yes	Yes	Unclear	Unclear	4
Balkanay 2015 II [17]	Yes	Unclear	Yes	Yes	Unclear	Unclear	4
Cho 2015 [12]	Yes	Sealed envelopes	Blinding of personnel	Yes	Unclear	Unclear	4
Djaiani G 2016 [18]	Yes	Sealed envelopes	Blinding of personnel	No	Yes	Unclear	3
Leino 2011 [19]	Yes	Sealed envelopes	No	Yes	Yes	Unclear	5
Li 2017 [14]	Yes	Sealed envelopes	Yes	No	Yes	Unclear	5
Liu 2016 [20]	Yes	Unclear	Unclear	Unclear	Unclear	Unclear personnel	1
Park 2014 [13]	Yes	Unclear	Unclear	Unclear	Unclear	Unclear	1
Shehabi 2009 [21] 2009	Yes	Unclear	Yes	No	Yes	Unclear	5
Ammar 2016 [22]	Yes	Unclear	Yes	Yes	Unclear	Unclear	4
Soliman 2016 [23]	Yes	Unclear	Yes	No	Unclear	Unclear	4

### Effect of Dexmedetomidine on incidence of AKI, and mortality

**Fig. 2:**
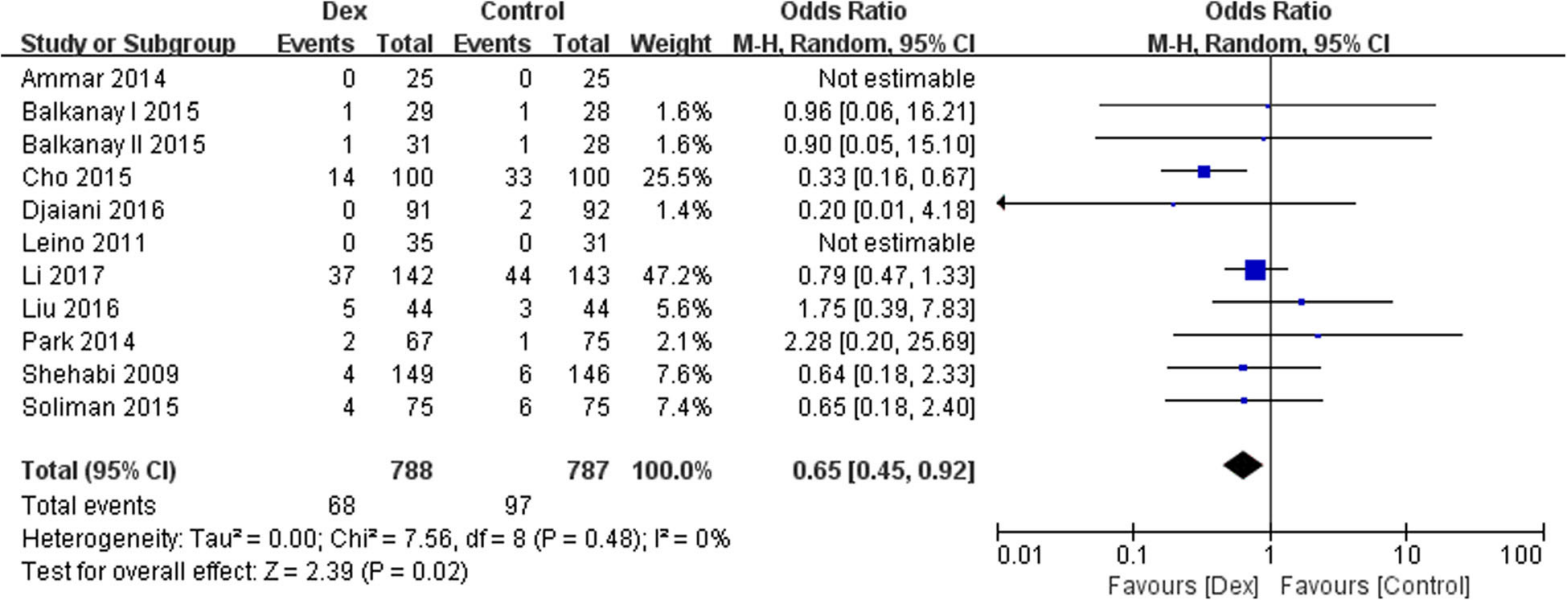
Dexmedetomidine (Dex) reduced the incidence of acute kidney injury

**Fig. 3:**
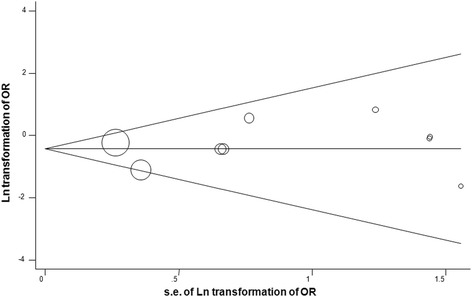
Funnel plot assessment of potential publication bias

The outcome of AKI was reported in 1575 study participants, and the overall incidence was 10.48% (dexmedetomidine group, 68/788; control group, 97/787). The postoperative incidence of AKI was significantly reduced by dexmedetomidine (10 studies with 11 comparision; OR, 0.65; 95% CI, 0.45–0.92; *P* = 0.02; I^2^ = 0.0%; Fig. 2). Different analysis method (Mantel-Haenszel or Inverse Variance) or different summary statistics (RR vs OR vs RD) was listed in Table 4.There was no evidence of significant publication bias (Begg’s test, *P* = 0.22; Egger’s test, *P* = 0.32; Fig. 3).

**Table 4:** Different analysis method and summary statistics for the incidence of acute kidney injury

Analysis method	OR	95%CI	I^2^	*P*	RD	95%CI	I^2^	*P*	RR	95%CI	I^2^	*P*
Mantel-Haenszel	0.65	0.45,0.92	0%	0.02	−0.02	−0.04,0.01	46%	0.28	0.72	0.54,0.95	0%	0.02
Inverse Variance	0.65	0.45,0.92	0%	0.02	−0.01	−0.04,0.01	21%	0.22	0.72	0.54,0.95	0%	0.02

*Abbreviations*: *OR* Odds ratio, *RR* Risk ratio, *RD* Risk difference, *CI* Confidence interval

Subgroup analyses for the potential sources of heterogeneity were listed in Table 5. We divided study participants into 11 groups according to different characteristics such as age(year, ≥60 versus <60), proportion of male (≥60% versus <60%), proportion with diabetes (≥25% versus <25%), CPB duration(min, ≥100 versus <100), statin use(≥60% versus <60%), loading dose (use or not), continuous infusion dosing (low versus high), controlled type (placebo versus nonplacebo), administration timing (pre/intraoperative versus postoperative), surgical type (CABG only versus combined) surgery, JADAD score (≥3 versus <3). Overall, no significant differences existed in the incidence of AKI (Table 5).

**Table 5:** Subgroup analyses for the potential sources of heterogeneity

Subgroup	Endpoint	No. of Comparisons	OR WMD	95% CI	*P* Value	I^2^	P_Difference_ Value
1. Age(years)	AKI	9	0.64	0.41~ 1.01	0.06	43.8%	0.18
≥ 60		5	0.54	0.31~ 0.94	0.03	32%	
< 60		4	1.12	0.45~ 2.79	0.81	0%	
2. Gender(Male%)	AKI	9	0.64	0.41~ 1.01	0.06	0%	0.91
≥ 60		4	0.70	0.28~ 1.74	0.45	47%	
< 60		5	0.75	0.46~ 1.20	0.22	0.0%	
3. Previous DM (%)	AKI	8	0.64	0.41 ~ 1.01	0.22	0%	0.86
≥ 25		4	0.75	0.48 ~ 1.18	0.22	0%	
< 25		4	0.68	0.21 ~ 2.14	0.51	49%	
4.CPB duration(minutes)	AKI	7	0.65	0.38 ~ 1.14	0.13	0%	0.59
≥ 100		3	0.61	0.27 ~ 1.36	0.22	60%	
< 100		4	0.85	0.34 ~ 2.15	0.73	0%	
5.Statin (%)	AKI	9	0.64	0.41 ~ 1.01	0.06	43.8%	0.18
≥ 60		5	0.54	0.31~ 0.94	0.03	32%	
< 60		4	1.12	0.45 ~ 2.79	0.81	0%	
6.Loading dose use	AKI	10	0.65	0.45 ~ 0.92	0.02	0%	0.86
Yes		4	0.72	0.24 ~ 2.10	0.54	0%	
No		6	0.64	0.40 ~ 1.02	0.06	16%	
7. Continuous infusion	AKI	9	0.61	0.42 ~ 0.88	0.008	68.3%	0.08
≥ 0.1 μg/kg/h		6	0.76	0.49~ 1.18	0.22	0%	
< 0.1 μg/kg/h		3	0.37	0.19 ~ 0.72	0.003	0%	
8. Control drugs	AKI	11	0.65	0.45 ~ 0.92	0.02	0%	0.33
Placebo		7	0.60	0.40 ~ 0.89	0.01	2%	
Others		4	0.96	0.40 ~ 2.29	0.93	0%	
9. Dex administration	AKI	11	0.65	0.45 ~ 0.92	0.02	0%	0.21
Pre/Intraoperation		8	0.59	0.40 ~ 0.87	0.007	0%	
Postoperation		3	1.11	0.45 ~ 2.74	0.83	0%	
10. Surgical procedures	AKI	11	0.65	0.45 ~ 0.92	0.02	0%	0.87
CABG or Aortic surgery		4	0.72	0.24 ~ 2.16	0.56	0%	
Combined		7	0.65	0.38 ~ 1.14	0.13	33%	
11. JADAD score	AKI	11	0.65	0.45 ~ 0.92	0.02	65.7%	0.09
≥ 3		9	0.59	0.41 ~ 0.86	0.006	0%	
< 3		2	1.88	0.53 ~ 6.73	0.33	0%	

*Abbreviations*: *AKI* Acute kidney injury, *OR* Odds ratio, *CI* Confidence interval, *DM* Diabetes mellitus, *CPB* Cardiopulmonary bypass, *Dex* Dexmedetomidine, *CABG* Coronary artery bypass graft

Sensitivity analysis excluding each included study at a time revealed that the Cho 2015 study was inconsistent with the direction and size of the overall AKI- reducing effect of dexmedetomidine (*P* = 0.34),and the other studies were consistent with the direction and size of the overall AKI- reducing effect of dexmedetomidine (P for all <0.04).

**Fig. 4:**
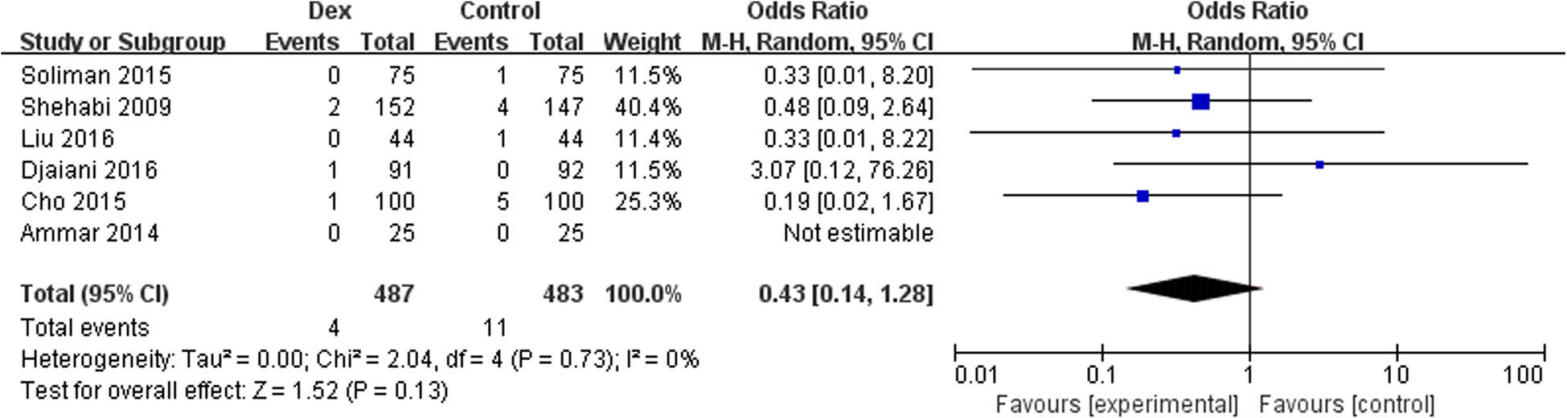
Forest plot for mortality

The outcome of mortality was reported in 970 study participants, and the overall incidence was 1.5% (dexmedetomidine group, 4/487; control group, 11/483). There were no statistically significant reduction for mortality owing to perioperative dexmedetomidine (6 studies; OR, 0.43; 95% CI, 0.14–1.28; *P* = 0.13; I^2^ = 0.0%; Fig. 4).

### Effect of Dexmedetomidine on MV duration, ICU stay and hospital stay

**Fig. 5:**
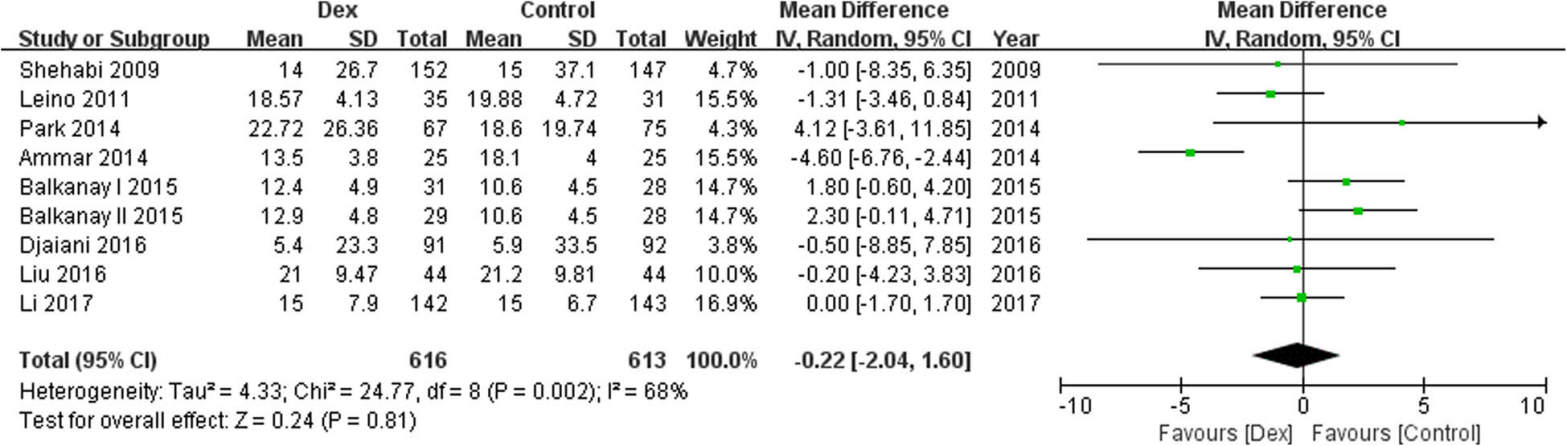
Forest plot for mechanical ventilation duration

**Fig. 6:**
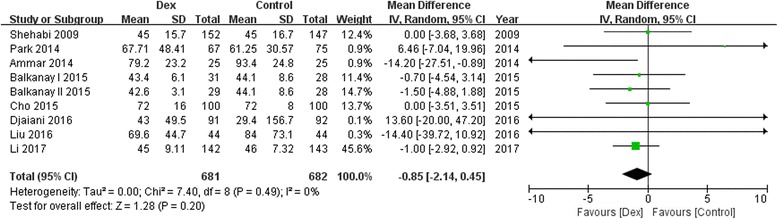
Forest plot for intensive care unit stay

**Fig. 7:**
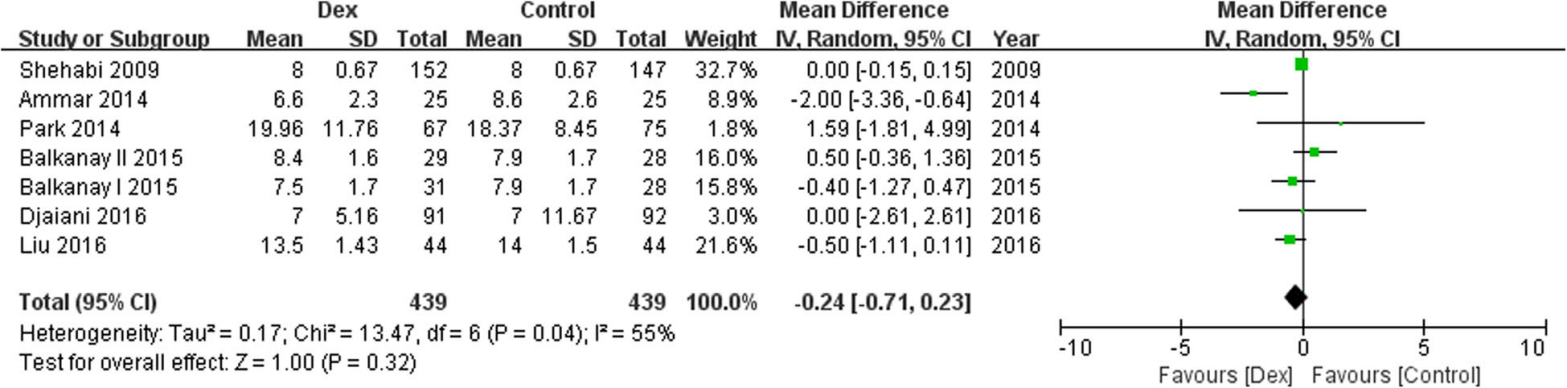
Forest plot for hospital length of stay

A trend toward reduction of postoperative MV duration(8 studies WMD, −0.22; 95%CI, −2.04 to 1.70; *P* = 0.81; I^2^ = 68%; Fig. 5), ICU stay(8 studies; WMD, −0.85; 95%CI, −2.14 to 0.45; *P* = 0.20; I^2^ = 0%; Fig. 6) and hospital stay (6 studies; WMD, −0.24; 95%CI, −0.71 to 0.23; *P* = 0.32; I^2^ = 55%; Fig. 7) by dexmedetomidine was observed, although there were not statistically significant.

## Discussion

In this meta-analysis of 10 RCTs involving 1575 adult patients undergoing cardiac surgery, we found that perioperative dexmedetomidine use was associated with a decrease in postoperative AKI risk. However, postoperative parameters including MV duration, ICU stay and hospital LOS appeared to be no significant decrease as a result of the dexmedetomidine use. To the best of our knowledge, this is the first meta-analysis evaluating the safety and efficacy of dexmedetomidine for the prevention of cardiac surgery associated AKI.

AKI is a common complication with an estimated incidence about 7% to 45% in adult cardiac surgery [24]. Small increases in postoperative serum creatinine levels after cardiac surgery have been reported to be associated with increased morbidity and mortality even if the renal function has returned to normal at discharge [25]. For this reason, strategies to lower the incidence of postoperative AKI are of high interest to clinicians.

Dexmedetomidine is widely used for perioperative anesthesia/analgesia, and may have a more profound renal protection by stabilizing the sympathetic system, exerting anti-inflammatory effects and attenuating ischemia/reperfusion (I/R) injury [10, 26]. In this meta-analysis, positive renoprotective effects were shown in 3 studies [12, 17, 22] and only 1 [12] study showed the prevention for the AKI. However, there were also controversial or negative studies pertaining to the effect of dexmedetomidine. Our analysis combining all these positive and negative studies showed a reduced incidence of AKI in association with the dexmedetomidine use. In view of the definition for AKI using conventional tests such as the blood urea nitrogen, serum creatinine levels, urine output quantity and creatinine clearance rate, it may result in delay in the timely detection of kidney injury and can lead to false-negative results, and dexmedetomidine for the prevention of AKI may be more effective than the current results.

In the included trials, dexmedetomidine was used with a loading dose (0.4μg/kg-1μg/kg) and continuous infusion (0.04–0.6μg/kg/h). Balkanay enrolled adult patients undergoing CABG found a significant difference between high dose group (8 μg/kg) and low dose group (4 μg/kg) for the 24th postoperative hour in the mean values of neutrophil gelatinase-associated lipocalin (NGAL) [17], indicating that dexmedetomidine had marked effects on renoprotection in a dose-dependent fashion. Our subgroup analyses showed that dexmedetomidine infusion without loading dose or at low continuous dose appeared to be safe and potentially efficacious by avoiding undesirable haemodynamic effects and was possibly more effective for renal-protection, although there was no significant difference (*P* = 0.86 and *P* = 0.08).To date, the optimal dose of dexmedetomidine to improve kidney function after cardiac surgery is unclear. The optimal dose of dexmedetomidine on postoperative renal events can’t be drew because of the lack of detailed patient data. Future large and well-designed randomized trails should explore the more appropriate dose of dexmedetomidine to maximize its renal protective effect with less side effects affecting prognosis.

The timing of dexmedetomidine administration in relation to cardiac surgery is emerging as an important consideration. In 6 of 10 included trials [12, 14, 17, 19, 22, 23], dexmedetomidine was used in a preemptive strategy, and early intervention of dexmedetomidine before the cardiopulmonary bypass seems to be critical for its organ-protective effect against I/R injury [27]. Dexmedetomidine pretreatment attenuated the I/R injury by reducing inflammatory response mediated by toll-like receptor4 expression [28, 29]. Our subgroup analyses indicated that dexmedetomidine was possibly more effective for renal-protection with pre/intraoperative administration compared with postoperative administration, but there was no significant difference (*P* = 0.21).Our findings do not provide a strong guidance on this question, and it merits further investigation. Future trials in this area would most likely be of greatest benefit.

Two recent expert consensus articles on postoperative AKI have been recently published, which discussed also new possible therapies/preventive measures [30, 31]. Our results was in keeping with one of the article conducted by M. Joannidis and colleagues, which showed dexmedetomidine was promising to reduce the rate of AKI, although no recommendation can be given on the basis of current data. Our subgroup analyses showed that dexmedetomidine was possibly effective for renal-protection compared with placebo but not against other treatments(*P* = 0.33). The advantages of dexmedetomidine compared with other anesthetics still call for further research.

Our analysis has several disadvantages. First, AKI in cardiac surgery is common and may have several different causes. It is difficult to establish a protective role for dexmedetomidine. We were unable to access individual patient data, so the influences of age, sex, and other confounding factors may be underestimated. Second, the definition of AKI was not uniform in the included trials. Third, sample size in each study is relatively low, so future large clinical studies were needed. Fourth, the exclusion of non-English studies may be inappropriate, however, the assessment of publication bias did not show statistical significance. Fifth, Bland [32] and Kwon & Reis [33] have argued that the statistical method of Hozo et al. may have limited their statistical ability to detect differences. When samples are not normally distributed. So the effect of dexmedetomidine may be overestimation, especially for negative findings. Last, perioperative dexmedetomidine might be of most benefit for certain patients who are at different stage of AKI, but most of the included study did not report the existed renal impairment before surgery.

## Conclusion

In summary, available evidence from the present meta-analysis suggests that perioperative administration of dexmedetomidine in adult cardiac surgery might reduce the incidence of AKI. Future trials are needed to be much larger and ascertain the optimal dose and, more importantly, the time of the dose, especially in patients with decreased kidney function at baseline.

## Abbreviations

AKI: Acute kidney injury
AKIN: Acute Kidney Injury Network
CI: Confidence interval
I/R: Ischemia/reperfusion
ICU: Intensive care unit
KDIGO: Kidney Disease Improving Global Outcomes
OR: Odds ratio
PRISMA: Preferred Reporting Items for Systematic Review and Meta-Analysis
RCTs: Randomized controlled trials
RIFLE: Risk–Injury–Failure–Loss–End-stage renal disease
WMD: Weighted mean difference
